# Supplementary figures and images for: SWI/SNF senses carbon starvation with a pH-sensitive low-complexity sequence
Source: eLife. 2022 Feb 7;11:e70344. doi: 10.7554/eLife.70344 (PMC8890752; doi:10.7554/eLife.70344)

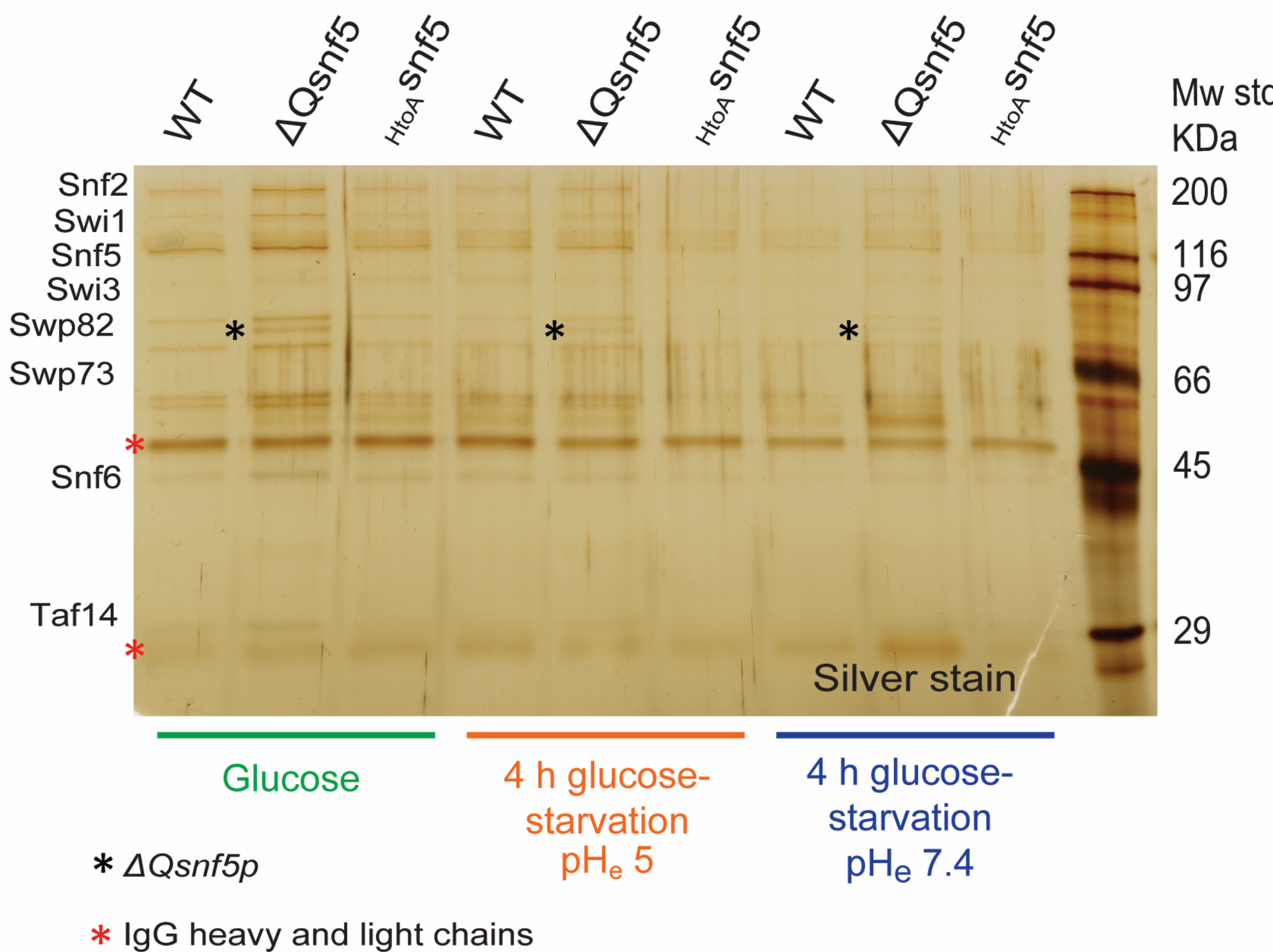

Supplement: Figure 1—figure supplement 7—source data 1. — Figure 1—figure supplement 7A: annotated. The endogenous SNF2 gene was tandem affinity purification (TAP)-tagged at the C-terminus and used to immunoprecipitate the SWI/SNF complex from WT, ΔQsnf5, or HtoAsnf5 strains either exponentially growing in glucose or after 4 hr acute carbon starvation in media titrated to pHe 5 or 7.4 (indicated at bottom). A silver stain of an SDS-PAGE analysis is shown. Figure 1—figure supplement 7A: unannotated. Silver-stained SDS-PAGE gel with no annotation. Figure 1—figure supplement 7B: annotated. Neither SNF5 nor its mutant alleles are degraded upon glucose starvation. Western blots of the TAP-tagged SNF5 alleles in various conditions (indicated at bottom). TAP-tagged ΔQ-snf5 runs at ~110 kDa, 288 amino acids smaller than WT (~160 kDa). An anti-glucokinase antibody was used as a loading control (bottom band at ~50 kDa). The SNF5-TAP bands are indicated by red boxes. Figure 1—figure supplement 7B: unannotated. Western blot with no annotation. [file elife-70344-fig1-figsupp7-data1.zip › Fig1Sup7A-source-annotated.pdf]

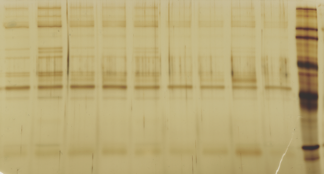

Supplement: Figure 1—figure supplement 7—source data 1. — Figure 1—figure supplement 7A: annotated. The endogenous SNF2 gene was tandem affinity purification (TAP)-tagged at the C-terminus and used to immunoprecipitate the SWI/SNF complex from WT, ΔQsnf5, or HtoAsnf5 strains either exponentially growing in glucose or after 4 hr acute carbon starvation in media titrated to pHe 5 or 7.4 (indicated at bottom). A silver stain of an SDS-PAGE analysis is shown. Figure 1—figure supplement 7A: unannotated. Silver-stained SDS-PAGE gel with no annotation. Figure 1—figure supplement 7B: annotated. Neither SNF5 nor its mutant alleles are degraded upon glucose starvation. Western blots of the TAP-tagged SNF5 alleles in various conditions (indicated at bottom). TAP-tagged ΔQ-snf5 runs at ~110 kDa, 288 amino acids smaller than WT (~160 kDa). An anti-glucokinase antibody was used as a loading control (bottom band at ~50 kDa). The SNF5-TAP bands are indicated by red boxes. Figure 1—figure supplement 7B: unannotated. Western blot with no annotation. [file elife-70344-fig1-figsupp7-data1.zip › Fig1Sup7A-source-unannotated.tiff]

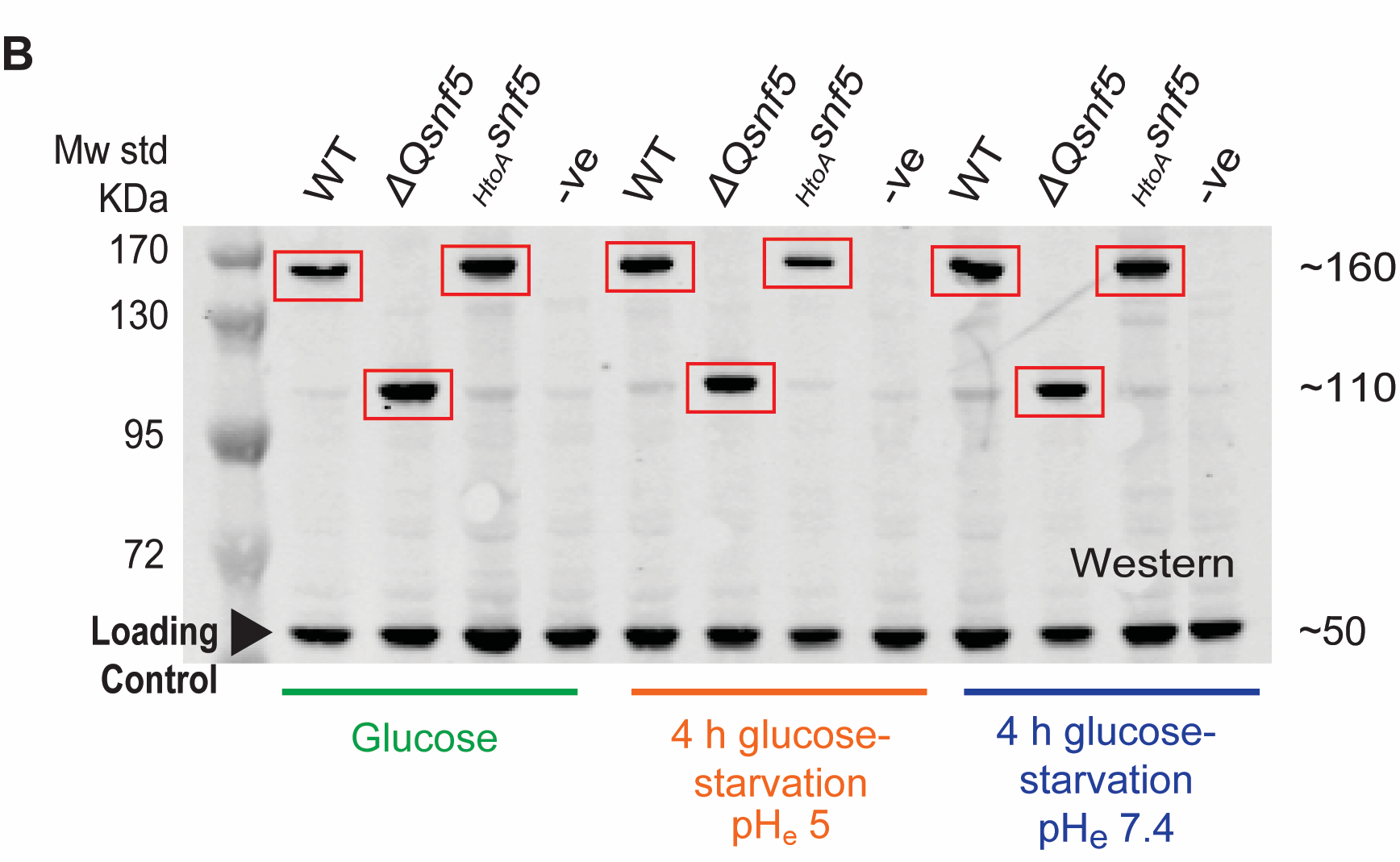

Supplement: Figure 1—figure supplement 7—source data 1. — Figure 1—figure supplement 7A: annotated. The endogenous SNF2 gene was tandem affinity purification (TAP)-tagged at the C-terminus and used to immunoprecipitate the SWI/SNF complex from WT, ΔQsnf5, or HtoAsnf5 strains either exponentially growing in glucose or after 4 hr acute carbon starvation in media titrated to pHe 5 or 7.4 (indicated at bottom). A silver stain of an SDS-PAGE analysis is shown. Figure 1—figure supplement 7A: unannotated. Silver-stained SDS-PAGE gel with no annotation. Figure 1—figure supplement 7B: annotated. Neither SNF5 nor its mutant alleles are degraded upon glucose starvation. Western blots of the TAP-tagged SNF5 alleles in various conditions (indicated at bottom). TAP-tagged ΔQ-snf5 runs at ~110 kDa, 288 amino acids smaller than WT (~160 kDa). An anti-glucokinase antibody was used as a loading control (bottom band at ~50 kDa). The SNF5-TAP bands are indicated by red boxes. Figure 1—figure supplement 7B: unannotated. Western blot with no annotation. [file elife-70344-fig1-figsupp7-data1.zip › Fig1Sup7B-source-annotated.tiff]

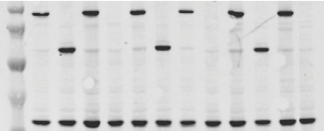

Supplement: Figure 1—figure supplement 7—source data 1. — Figure 1—figure supplement 7A: annotated. The endogenous SNF2 gene was tandem affinity purification (TAP)-tagged at the C-terminus and used to immunoprecipitate the SWI/SNF complex from WT, ΔQsnf5, or HtoAsnf5 strains either exponentially growing in glucose or after 4 hr acute carbon starvation in media titrated to pHe 5 or 7.4 (indicated at bottom). A silver stain of an SDS-PAGE analysis is shown. Figure 1—figure supplement 7A: unannotated. Silver-stained SDS-PAGE gel with no annotation. Figure 1—figure supplement 7B: annotated. Neither SNF5 nor its mutant alleles are degraded upon glucose starvation. Western blots of the TAP-tagged SNF5 alleles in various conditions (indicated at bottom). TAP-tagged ΔQ-snf5 runs at ~110 kDa, 288 amino acids smaller than WT (~160 kDa). An anti-glucokinase antibody was used as a loading control (bottom band at ~50 kDa). The SNF5-TAP bands are indicated by red boxes. Figure 1—figure supplement 7B: unannotated. Western blot with no annotation. [file elife-70344-fig1-figsupp7-data1.zip › Fig1Sup7B-source-unannotated.tiff]
